# Home-based circuit training improves blood lipid profile, liver function, musculoskeletal fitness, and health-related quality of life in overweight/obese older adult patients with knee osteoarthritis and type 2 diabetes: a randomized controlled trial during the COVID-19 pandemic

**DOI:** 10.1186/s13102-024-00915-4

**Published:** 2024-06-03

**Authors:** Sameer Badri Al-Mhanna, Alexios Batrakoulis, Mahaneem Mohamed, Nouf H. Alkhamees, Bodor Bin Sheeha, Zizi M. Ibrahim, Abdulaziz Aldayel, Ayu Suzailiana Muhamad, Shaifuzain Ab Rahman, Hafeez Abiola Afolabi, Maryam Mohd Zulkifli, Muhammad Hafiz bin Hanafi, Bishir Daku Abubakar, Daniel Rojas-Valverde, Wan Syaheedah Wan Ghazali

**Affiliations:** 1 Department of Physiology, School of Medical Sciences, Universiti Sains Malaysia, Kubang Kerian, Kelantan, Malaysia; 2 Department of Physical Education and Sport Science, School of Physical Education, Sport Science and Dietetics, University of Thessaly, Trikala, Greece; 3 Department of Rehabilitation Sciences, College of Health and Rehabilitation Sciences, Princess Nourah bint Abdulrahman University, P.O. Box 84428, Riyadh, 11671 Saudi Arabia; 4 Department of Physical Therapy for Surgery, Faculty of Physical Therapy, Cairo University, Cairo, Egypt; 5 Department of Exercise Physiology, King Saud University, Riyadh, Saudi Arabia; 6 Exercise and Sports Science Program, School of Health Sciences, Universiti Sains Malaysia, Kubang Kerian, Kelantan, Malaysia; 7 Department of Orthopaedic, Hospital University Sains Malaysia, Universiti Sains Malaysia, Kubang Kerian, Kelantan Malaysia; 8 Department of General Surgery, School of Medical Sciences, Hospital Universiti Sains Malaysia, Kubang Kerian, Kelantan, Malaysia; 9 Department of Family Medicine, School of Medical Sciences, Universiti Sains Malaysia, Kubang Kerian, Kelantan, Malaysia; 10 Rehabilitation Medicine Unit, School of Medical Sciences, Universiti Sains Malaysia, Kubang Kerian, Kelantan, Malaysia; 11 Department of Human Physiology, Federal University Dutse, Dutse, Jigawa State Nigeria; 12 Centro de Investigación y Diagnóstico en Salud y Deporte, Escuela Ciencias del Movimiento Humano y Calidad de Vida Universidad Nacional de Costa Rica, Heredia, Costa Rica; 13 Center for Global Health Research, Saveetha Medical College and Hospitals, Saveetha Institute of Medical and Technical Sciences, Chennai, Tamil Nadu 602105 India

**Keywords:** Quality of life, Aerobic exercise, Cardiometabolic health, Muscular fitness, Metabolic syndrome, Resistance training

## Abstract

**Background:**

There is strong evidence showing the association between obesity, type 2 diabetes mellitus (T2DM), and knee pain resulting from osteoarthritis. Regular exercise has been reported as a foundational piece of the preventive therapy puzzle for knee osteoarthritis (KOA) patients. Nonetheless, evidence-based exercise protocols for people with comorbidities, such as obesity, T2DM, and KOA are limited. Therefore, the present trial aimed to assess the effectiveness of a 12-week home-based circuit training (HBCT) protocol on various indices related to cardiometabolic health, musculoskeletal fitness, and health-related quality of life (HRQoL) among overweight/obese older adult patients with KOA and T2DM during the COVID-19 lockdown.

**Methods:**

This is a randomized controlled trial study registered at the National Medical Research Register (ID: RSCH ID-21-01180-KGTNMRR ID-21-02367-FUM) and obtained approval on December 9, 2021. Seventy overweight or obese patients with KOA and T2DM (62.2 ± 6.1 years; 56% female) were randomly assigned to the intervention group (*n* = 35, HBCT) or the no-exercise control group (*n* = 35, CON). HBCT performed a 12-week progressive protocol (seven exercises; 15–30 repetitions per exercise, 1 min passive rest between exercises; 2–4 rounds per session; 20–60 min total session duration). Blood samples were collected, and assays were performed to assess the lipid profile, liver function, and fasting blood glucose (FBG). In addition, the 30-s Chair Stand Test (30CST) was used to evaluate lower body muscular strength and endurance while the Timed Up and Go (TUG) test was used to evaluate lower limb function, mobility, and the risk of falls for all the participants. HRQoL was assessed using the Osteoarthritis Knee and Hip Quality of Life (OAKHQoL). All the assessments were conducted at pre-, mid-, and post-training stages during the application or practice of the exercise protocol, rather than during the training sessions themselves.

**Results:**

HBCT significantly reduced total cholesterol (TC), triglycerides (TG), low-density lipoprotein cholesterol (LDL-C), aminotransferase, alanine aminotransferase, FBG and knee pain (*p* < 0.05). Furthermore, HBCT induced meaningful increases in high-density lipoprotein (HDL-C), lower body muscular strength, endurance, function, mobility, and HRQoL in overweight/obese older adults with T2DM and KOA (*p* < 0.05).

**Conclusion:**

The present outcomes recommend that an injury-free HBCT program may improve various indicators related to cardiometabolic health, musculoskeletal fitness, and HRQoL in elderly with overweight/obesity, T2DM and KOA. These findings offer valuable insights for clinicians and practitioners seeking evidence-based exercise interventions tailored for patients managing substantial metabolic and musculoskeletal health challenges in clinical practice.

## Introduction

The global healthcare systems are significantly influenced by the pervasive presence of obesity and its adverse effects [1]. Since 1980, the prevalence of obesity has shown a notable rise, as indicated by the extensive examination of data from 195 countries conducted by the Worldwide Burden of Disease research [2, 3]. The rise in obesity rates worldwide has been accompanied by a corresponding surge in the occurrence of type 2 diabetes mellitus (T2DM) [2, 3]. Interestingly, several studies have demonstrated a correlation between the deterioration of knee pain and the existence of one or multiple comorbid conditions, including obesity and T2DM [4–6].

It is worth mentioning that a unit increase in body mass index (BMI) can elevate the likelihood of developing knee osteoarthritis (KOA) by 15% [7]. Strong evidence shows a high correlation between T2DM and KOA [8, 9], and this correlation has been linked to lower levels of physical activity and self-efficacy for strength training [10]. One of the primary underlying mechanisms causing KOA in T2DM patients is assumed to be the increased mechanical strain on weight-bearing joints, particularly the knees since both diseases share overweight and obesity as significant shared risk factors. Compared to non-diabetic adults, patients with T2DM and KOA often have poorer muscular strength and lower leg skeletal mass [11, 12], and the impaired muscular function may result in insulin resistance [13, 14]. Importantly, the literature also implies that quadriceps strength is crucial for comprehending the variety and importance of the results of a 30-s chair stand test (30CST) [15]. Improving a measure of lower limb strength is one of the primary drivers behind persons with chronic, acute, and devastating KOA seeking exercise is an improvement in leg strength and endurance, lower limb function, and mobility [16–18].

Engaging in regular exercise enhances both muscle strength and mass [19], as well as reducing the levels of inflammatory cytokines and providing protection to the knee joint [20]. According to a prior investigation by Juhl et al. (2014), the most effective exercise regimens for treating patients with KOA should prioritize lower limb functionality and cardiovascular endurance. However, Bennell, Hinman [21] suggested that an effective strategy for managing the various complexities associated with OA consists of integrating both resistance and aerobic exercise, which also seems as one of the most popular and beneficial types of exercise for improving numerous cardiometabolic health and musculoskeletal fitness markers among individuals with a BMI ≥ 25 kg/m^2^ [22–29]. However, individuals with excessive weight demonstrate low adherence and high attrition rates to movement-based programs due to BMI perception and body image dissatisfaction related to poor functional capacity as well as impaired musculoskeletal and mental health [30, 31].

Circuit training (CT) is an effective training program for improving cardiorespiratory and musculoskeletal fitness, including a group of muscle-strengthening exercises of all the major muscle groups that are completed consecutively with minimal or no rest intervals. Also, aerobic-based exercises can be included, which has potential benefits for various chronic diseases, including KOA [32]. In this training program, each exercise is performed for a specified number of repetitions or for a set time seconds before a brief rest and moving onto the next exercise and therefore the participants’ heart rate can be elevated throughout the workout [33, 34]. Such a workout structure requires shorter rest periods compared to traditional strength training, resulting in significantly reduced overall workout time [35, 36] while considering attractive in the global exercise community [37].

Despite the positive exercise training-induced adaptations, the most effective exercise approach for patients with obesity, T2DM, and KOA is currently unclear. Thus, this pragmatic randomized controlled trial was conducted in a home-based setting, aiming to primarily assess the efficacy of a 3-month home-based circuit training (HBCT) protocol on (i) lipid homeostasis, (ii) liver function, (iii) fasting blood glucose (FBG), and (iv) musculoskeletal fitness among sedentary overweight/obese older adult patients with KOA and T2DM. The hypothesis posited that the intervention group (HBCT) would induce more pronounced positive changes in comparison to the control group (CON).

## Methods

### Study design

This is a randomized controlled trial study registered at the National Medical Research Register (ID: RSCH ID-21-01180-KGTNMRR ID-21-02367-FUM) https://nmrr.gov.my/submission/e6b92c2d-867f-4304-a87e-0510f4216a7f.

**Fig. 1:**
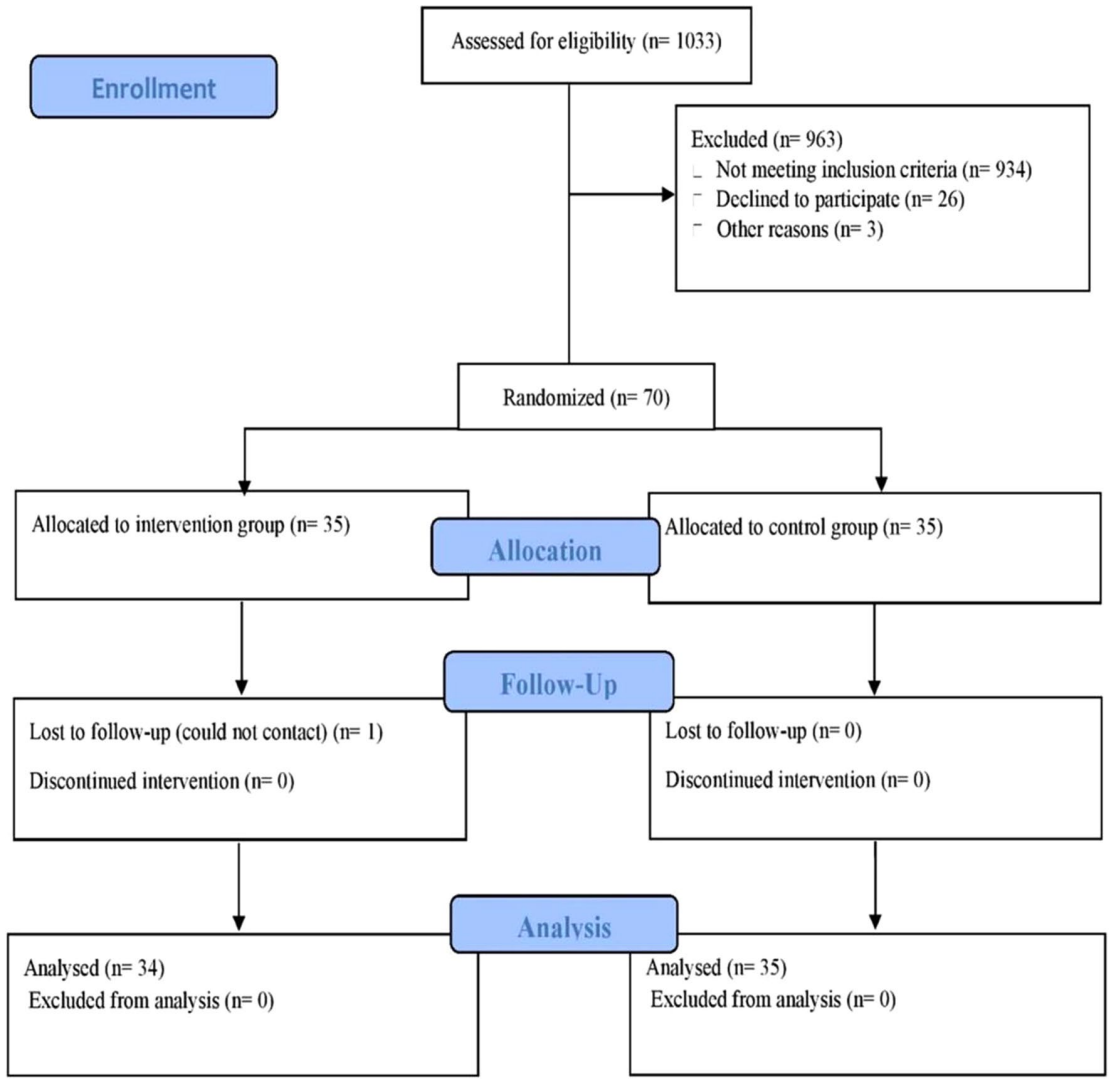
Consort flow diagram of the study

The study was conducted at the Physiology Laboratory of the School of Medical Sciences at the University Sains Malaysia (USM) from September 13, 2021 until September 12, 2022. Ethical approval was obtained from the Human Research Ethics Committee of USM before the commencement of the study (approval code: JEPeM 21,050,374). This study adhered to the CONSORT guidelines. Initially, 162 patients were approached via posters distributed in the Orthopedics Clinic at USM Hospital and were screened by phone. Of these, 70 met the inclusion criteria and were randomly recruited into the study. Pre-training assessments were conducted before the randomization to determine participants’ eligibility. Written informed consent was obtained from all participants prior to the study. Patients were randomly divided into two groups through a computer-generated random allocation sequence, conducted independently by a statistician using simple randomization (http:/www.randomization.com). Additionally, the statistician was blinded and remained unaware of the group’s allocation. Similarly, the assessors were kept blinded, with no knowledge of both the study objectives and the random allocation of patients to different study groups throughout the entire process. Fig 1 illustrates the CONSORT flow diagram of the study.

### Participants

The sample size for this study was calculated based on repeated measure ANOVA within-between factors using a preliminary power analysis (G*Power 3.1.9.2) with type I error of 0.05, power of study 80%, number of groups of 2, number of measurements of 3, a medium effect-size of 0.25 correlation between the repeated measures of 0.5, non-sphericity correction ε of 1, and considering a dropout of 20% as reported in previous training studies with overweight/obese individuals investigating similar outcome measures [23, 26, 28, 29, 31, 38]. A total of 35 participants in each group were required for this study. This study involved 70 patients meeting the inclusion criteria as follows: (i) age > 55 years, (ii) diagnosed with KOA with Kellgren-Lawrence criteria grades 2 and 3, indicating moderate KOA, which was based on radiological assessments conducted by a traumatologist, (iii) two and three KOA (unilateral or bilateral), (iv) chronic knee pain for more than three months, (v) T2DM based on fasting plasma glucose > 7.0 mmol·L-1 and glycated hemoglobin (HbA1c) > 6.5%, (vi) overweight or obesity (BMI ≥ 25 kg/m^2^), (vii) receiving the standard treatment (all patients were taking diabetes medications), and (viii) providing a certificate of a negative COVID-19 diagnostic test (PCR or rapid test). Patients were excluded from the study if during the intervention they demonstrated (i) secondary KOA, (ii) acute knee pain, (iii) changes in medication, supplementation, and/or diet, (iv) changes in habitual physical activity, (v) intraarticular hyaluronic acid injection treatment within one year, (vi) smoking, (vii) dementia or any psychiatric diseases, (viii) adherence to less than 90% of the total prescribed exercise sessions, or (ix) they tested positive for COVID-19. The Charlson Comorbidity Score (CCS) was used to evaluate patients’ health status, examining 17 chronic diseases with assigned weighted scores or values index [39]. Patients’ CCS was used to group the comorbidity into the following groups: 0: none, 1–2: mild, 3–4: moderate, and ≥ 5: severe [40, 41]. Following the provision of informed written consent, patients were instructed to abstain from any additional forms of exercise and to uphold their prevailing habitual physical activity levels and dietary patterns over a 3-month intervention period. Consequently, participants’ logbooks were scrutinized at each visit to verify the absence of alterations in nutritional behavior and physical activity patterns. CON group followed standard treatment (diabetes medications) without engaging in any structured exercise throughout the intervention. Table 1 shows participants’ characteristics.

**Table 1:** Participants’ baseline characteristics

Variable	CON (*n* = 35)	HBCT (*n* = 35)	*p*-value
*Gender, n (%)*			0.910 ^a^
Male	16 (51.6)	15 (48.4)	
Female	19 (48.7)	20 (51.2)	
Age (yrs)	61.70 (5.20) ^c^	62.60 (6.90) ^c^	0.540
Weight (kg)	80.80 (15.89) ^c^	80.6 (10.42) ^c^	0.930
Height (m)	1.57 (0.86) ^c^	1.58 (0.88) ^c^	0.720
BMI (kg/m^2^)	32.81 (5.60) ^c^	32.41 (4.30) ^c^	0.880
Comorbidity score	6.10 (1.10) ^c^	5.70 (1.10) ^c^	0.370
*T2DM medication, n (%)* Metformin Metformin & Gliclazide Actrapid	21 (52.5) 12 (48) 2 (40)	19 (47.5) 13 (52) 3 (60)	0.870 ^a^
FBG (mmol/L)	8.19 (2.91) ^c^	8.59 (2.17) ^c^	0.679
*Blood Lipid Profile*			
HDL-C (mmol/L)	1.24 (0.27) ^c^	1.15 (0.24) ^c^	0.170
TG (mmol/L)	2.51 (0.71) ^c^	2.86 (0.88) ^c^	0.071
LDL-C (mmol/L)	2.79 (1.20) ^c^	3.21 (0.99) ^c^	0.075
TC (mmol/L)	4.52 (1.16) ^c^	4.86 (1.14) ^c^	0.218
*Liver Function*			
Total protein	11.17 (3.93) ^c^	10.71 (5.25) ^c^	0.678
AST (U/L)	27.91 (13.38) ^c^	25.06 (8.28) ^c^	0.292
ALT (U/L)	36.37 (21.11) ^c^	33.03 (10.19) ^c^	0.342
ALP (U/L)	93.20 (24.96) ^c^	89.06 (27.85) ^c^	0.517
*Musculoskeletal Fitness*			
30CST (repetitions)	7.46 (3.34) ^c^	5.82 (1.37) ^c^	0.054
TUG (s)	11.42 (3.18) ^c^	12.66 (2.36) ^c^	0.072
*Pain score (VAS)*	5.62 (1.68) ^c^	5.58 (1.93) ^c^	0.931
*Comorbidity score*	6.14 (1.10) ^c^	5.70 (1.10) ^c^	0.370
*HRQoL*			
Social function	17.63 (9.20) ^c^	13.76 (7.19) ^c^	0.067
Mental health	32.63 (17.16) ^c^	34.85 (22.17) ^c^	0.642
Social support	17.54 (8.80) ^c^	16.52 (7.43) ^c^	0.607
Pain	19.51(8.63) ^c^	20.21 (9.0) ^c^	0.746
Physical activity function	33.89 (16.01) ^c^	35.85 (19.01) ^c^	0.140

^a^Chi-square test, ^c^mean (SD). 30CST, 30-s chair stand test; ALP, alkaline phosphatase; ALT, alanine transaminase; AST, aspartate transaminase; BMI, body mass index; FBG, fasting blood glucose; HDL-C, high-density lipoprotein cholesterol; HRQoL, health-related quality of life; LDL-C, low-density lipoprotein cholesterol; TC, total cholesterol; TG, triglycerides; TUG, timed up and go test

### Exercise protocol

**Fig. 2:**
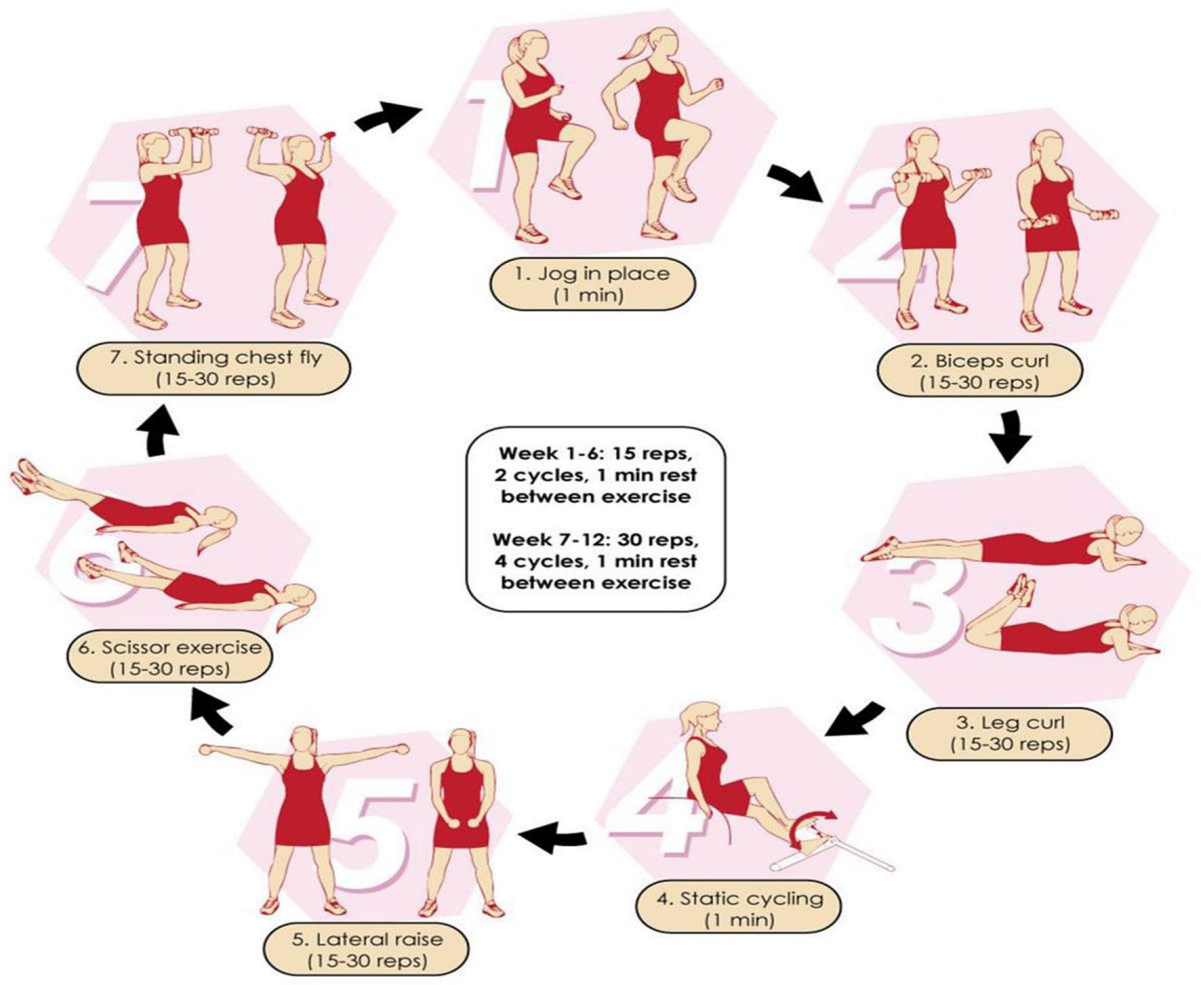
Home-based circuit training program

Patients were encouraged not to change their habitual physical activity levels during the study. Any deviation from the protocol was considered a reason for exclusion. During regular follow-up, participants were asked whether they had engaged in any activity that might be interpreted as interference with the intervention. Participants in the exercise group performed HBCT training three times per week on non-consecutive days for 12 weeks. However, the first session was conducted at USM Hospital and participants were instructed how to perform the prescribed exercises in a correct form. The HBCT protocol was adapted from a previous study [42] and is shown in Fig. 2. Progressive overloading was applied for the safety of participants, since they were sedentary overweight/obese adults with KOA and T2DM as well as to allow continuous progress to occur. In each session, participants performed seven exercises (two aerobic- and five resistance-based) in a circuit fashion, using bodyweight movements and adjustable dumbbells for varied weights, activating all the major muscle groups. In weeks 1–6, participants executed 15 repetitions for 2 rounds with 1 min passive rest between exercises and rounds. In weeks 7–12, participants executed 30 repetitions for 4 rounds with the same rest as prescribed in weeks 1–6. Each round lasted 10–15 min and the total session duration was 20–60 min, aiming to help participants adapt gradually to increasing training volume. Such an exercise programming approach aimed to limit potential training-related injuries, overreaching and overtraining while providing an engaging and inclusive exercise experience in a real-world setting. A tutorial video was developed and provided to participants to ensure a correct demonstration of all prescribed exercise techniques with proper form at a controlled, moderate speed. For resistance-based exercises, participants were encouraged to use a comfortable weight at the beginning of the study and progressed to heavier weights that allowed them to complete the prescribed number of repetitions at each exercise station. A 5-min warm-up and a 5-min cool-down period were applied in all sessions. The rate of perceived exertion (RPE) was self-recorded using the Borg scale ranging from 6 (rest) to 20 (maximal) [43]. RPE values were recorded for each round and mean exertion was calculated. Participants were advised to adjust the magnitude of their effort progressively during the 12-week intervention (weeks 1–6: RPE 11–13; weeks 7–12: RPE 14–16). A logbook was given to the participants to record their adherence to the prescribed exercise sessions and the RPE values per session. The logbook was checked through weekly follow-up via telephone calls and during each visit. Furthermore, a text message was sent to HBCT patients on a weekly basis regarding inquiries about the exercise routine.

### Assessment procedures

**Fig. 3:**
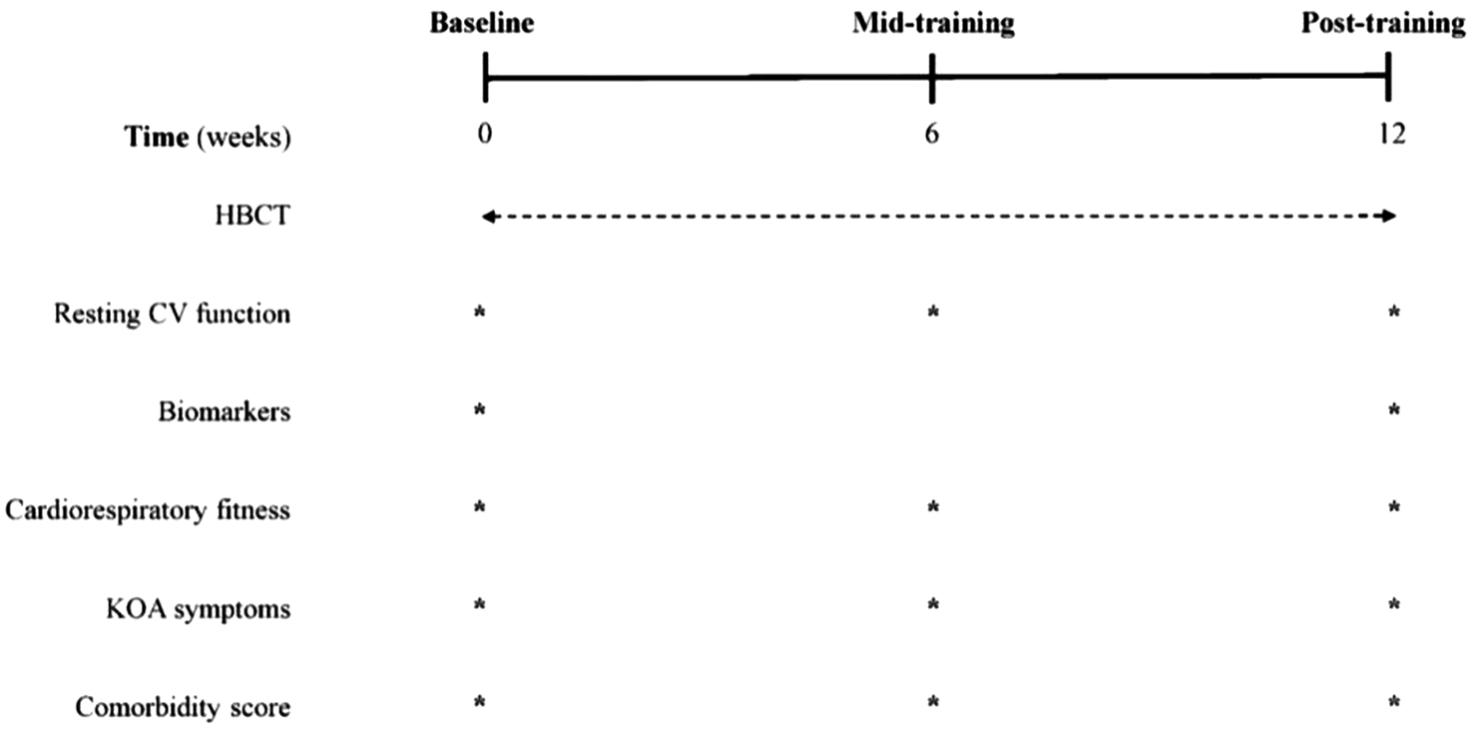
Experimental flow chart. HBCT; home-based circuit training; CV, cardiovascular function; KOA, knee osteoarthritis

All patients were instructed to avoid consuming caffeinated beverages and strenuous exercise 24 h before the first visit. A total of three visits were performed and all outcomes were assessed through the three visits (baseline, weeks 6 and 12) in the morning (07:00–09:00 a.m.) after an overnight fast (Fig. 3). All measurements were performed at USM Hospital. During the first visit (baseline), assessments were carried out for body mass index (BMI), FBG, lipid profile, liver function parameters as well as the level of pain using Visual Analogue Scale (VAS) score. Comorbidity was assessed using the Charlson Comorbidity Score [41]. In addition, the 30-s Chair Stand Test (30CST) was used to evaluate lower body muscular strength and endurance while the Timed Up and Go (TUG) test was used to evaluate lower limb function, mobility, and the risk of falls for all the participants. Health-related quality of Life (HRQoL) was assessed using the Osteoarthritis Knee and Hip Quality of Life (OAKHQoL). During the second (mid-testing) and third (post-testing) visits at week 6 and 12, respectively, similar assessments to those conducted at baseline were carried out, except for biomarkers that were not assessed at week 6. They were not given access to the randomization sequence. Similarly, the statistician responsible for data analysis was also blinded and unaware of the group allocation.

#### Biomarkers

Blood samples and assays were carried out according to standard procedures as previously described [28]. In brief, the blood samples (10 ml) were drawn from the participants after 12 h of fasting, put into a heparinized sterile tube, and kept on ice until processing. The blood was centrifuged at 1500 rpm for 15 min at 4 °C. The separated serum was stored at − 80 °C until further analysis. Commercially available kits (Human) were used for measuring TG (#10,724), TC (#10,028), and HDL (#10,018) on an automated clinical chemistry analyzer HumaStar 200 (Human Diagnostics, Wiesbaden, Germany). FBG was determined using a commercially available ELISA kit (#KA4088, Abnova Corporation, Taipei, Taiwan) as previously reported [28]. Low-density lipoprotein cholesterol (LDL-C) concentration was calculated according to the equation LDL = TC − HDL − (TG / 5) [44]. All assays were performed in duplicates on the same day. The inter- and intra-assay coefficients of variability for all assays ranged from 6.1 to 10.8%, 5.3–9.7%, and from 4.9 to 8.6%, respectively.

#### Musculoskeletal fitness

The 30CST and the TUG tests were administered to the participants under the supervision of assessors who provided standard instructions. During the 30CST, participants began in a seated position, and upon the command “go,” they repeatedly stood up and sat down as quickly as possible for 30 s. The assessors recorded the total number of repetitions completed by the participants as the standard clinical outcome for the 30CST [45]. On the other hand, during the TUG test, participants started in a seated position on a chair. Following the command “go,” they rose from the chair, walked three meters comfortably and safely, made a 180° turn, returned to the chair, and sat down again. The assessors used a stopwatch to record the total time taken to perform the TUG test as the standard clinical outcome for this assessment [46].

#### Health-related quality of life

The osteoarthritis knee and hip quality of life (OAKHQoL) questionnaire was used as a validated tool to assess how KOA specifically affects the HRQoL of participants [47]. This questionnaire considers specific criteria relevant to the HRQoL of individuals with KOA, such as social support, sleep, medication side effects, feelings of embarrassment, use of public transportation, difficulty in movement after prolonged stillness, and sexuality. It comprises 31 items categorized into five domains: physical activity, pain, mental health, social function, and social support. The preliminary testing showed the reliability of the five dimensions to be satisfactory (intraclass correlation coefficients: 0.70–0.85). Each item within the domains was assessed on a numerical rating scale from 0 to 10. The final scores were calculated as the average of all the item scores within their respective domains, ranging from 0 to 10 [48].

#### Pain

A 100-mm horizontal VAS was employed to assess pain levels. The pain VAS was defined as painless on the left and worst pain on the right of the horizontal scale. Following a comprehensive explanation of how to effectively utilize the pain VAS, participants were instructed to independently mark their perceived level of pain on the scale. The resulting pain VAS score was subsequently determined by measuring the distance in millimeters from the leftmost point of the scale to the participant’s marked location [49].

### Statistical analysis

The Shapiro-Wilk test was used to verify data normality. Participants’ baseline characteristics were compared using an independent t-test for normally distributed data and the Mann-Whitney test for not normally distributed data. A mixed ANOVA model with a simple main effect analysis of time/group was applied to give estimates based on group and time, and detect between time points across the groups. Effect sizes were calculated using eta squared (*η*^2^) and were interpreted as small, medium-sized, and large for values 0.01–0.05, 0.06–0.13, and ≥ 0.14, respectively [50]. A value of *p* < 0.05 was considered to be statistically significant. Statistical analysis was carried out using the SPSS 27.0 software (IBM Corp., Armonk, NY, USA). Results were presented as mean difference (MD), 95% confidence intervals (CI), *η*^2^ and *p* values.

## Results

Out of 1033 patients assessed for eligibility, 963 (93%) were excluded. Of these, 934 (96%) did not meet the inclusion criteria, while 26 (3%) declined participation. The remaining three exclusions (0.3%) were due to other reasons, such as family and work commitments. Therefore, a total of 69 participants (HBCT, *n* = 34; CON, *n* = 35) completed the study and the attendance rate for HBCT was 91.7% (Fig. 1). One patient in HBCT dropped out due to loss of follow-up (dropout rate: 1.4%) whereas patients in CON attended all three visits. No injuries or adverse effects were reported during the intervention. No changes were detected in habitual physical activity, eating habits, CCS score and medication use throughout the study.

As shown in Table 1, no differences were found in all variables between groups at baseline. HBCT showed significant improvements (*p* < 0.05) in HDL-C (+ 20%), LDL-C (-29.3%), TG (-38.8%), TC (-26.5%), and FBG (-19.3%) from baseline to post-training. AST, ALT, and total protein demonstrated meaningful reductions (*p* < 0.05) by 15.4%, 16.1% and 12.3% from baseline to post-training, respectively, but not ALP (Table 2).

**Table 2:** Pairwise comparisons between HBCT and CON in time for biomarkers

Variables	CON		HBCT	
	MD (95% CI)		MD (95% CI)	
	Pre vs. Post	*p*	Pre vs. Post	*p*
FBG (mmol/L)	-0.01 (-1.04, 1.02)	0.982	1.66 (0.98, 2.35)	< 0.001*
*Blood Lipid Profile*				
TC (mmol/L)	0.07 (-0.31, 0.45)	0.694	1.29 (0.76, 1.81)	< 0.001*
HDL-C (mmol/L)	0.10 (− 0.02, 0.210)	0.090	-0.23 (-0.36, -0.11)	< 0.001*
LDL-C (mmol/L)	-0.14 (-0.41, 0.13)	0.308	0.94 (0.58, 1.30)	< 0.001*
TG (mmol/L)	0.213 (-0.09, 0.51)	0.159	1.04 (0.76, 1.33)	< 0.001*
*Liver Function*				
Total protein	0.26 (-1.9, 2.43)	0.811	-1.32 (-2.44, -0.21)	0.022*
AST (U/L)	0.71 (-1.34, 2.77)	0.484	3.85 (1.72, 5.98)	0.001*
ALT (U/L)	-0.23 (-3.82, 3.37)	0.898	5.32 (1.48, 9.17)	0.008*
ALP (U/L)	0.03 (-0.03, 0.09)	0.348	-0.07 (-0.02, 0.16)	0.159

MD; mean difference, CI; confidence intervals; ALP, alkaline phosphatase; ALT, alanine transaminase; AST, aspartate transaminase; FBG, fasting blood glucose; HDL-C, high-density lipoprotein cholesterol; LDL-C, low-density lipoprotein cholesterol; TC, total cholesterol; TG, triglycerides. * denotes statistical significance (*p* < 0.05)

Table 3 shows the comparison within groups in time. For HBCT, significant improvements were observed in BMI (-4% to -5%), 30CST (+ 18–56%), TUG (+ 24–29%) and pain (-30% to -61%) at mid- (*p* = 0.002 − 0.001) and post-training (*p* < 0.001) compared to baseline, as well as from mid-training to post-training (*p* = 0.012 − 0.001) in 30CST and the pain score, but not in BMI and TUG. As for HRQoL, HBCT exerted meaningful alterations in pain (-22%) and physical activity function (+ 16%) at post-training compared to the baseline levels (*p* < 0.001), but not in social support, social function and mental health. For CON, there was no significant difference in BMI, 30CST, TUG, the pain score and HRQoL’s dimensions throughout the intervention.

**Table 3:** Pairwise comparisons between HBCT and CON in time for BMI, musculoskeletal fitness, and HRQoL.

Variables	CON						HBCT					
	MD (95% CI)		MD (95% CI)		MD (95% CI)		MD (95% CI)		MD (95% CI)		MD (95% CI)	
	Pre vs. Mid	*p*	Pre vs. Post	*p*	Mid vs. Post	*p*	Pre vs. Mid	*p*	Pre vs. Post	*p*	Mid vs. Post	*p*
BMI (kg/m^2^)	-0.28 (-0.91, 0.35)	0.802	-0.30 (-0.75, 0.15)	0.295	-0.02 (-0.57, 0.52)	0.950	1.25 (-1.02, 3.51)	0.002*	1.65 (1.25, 2.05)	< 0.001*	0.40 (-1.94, 2.75)	0.602
*Musculoskeletal Fitness*												
30CST (repetitions)	0.14 (-0.90, 1.19)	0.950	0.66 (-0.49, 1.80)	0.477	0.51 (-0.06, 1.09)	0.095	-2.24 (-2.98, -1.49)	< 0.001*	-3.27 (-4.36, -2.17)	< 0.001*	-1.03 (-1.86, -0.19)	0.012*
TUG (s)	-0.24 (-1.09, 0.61)	0.950	-0.70 (-2.01, 0.61)	0.560	-0.46 (-1.63, 0.71)	0.986	2.99 (2.29, 3.69)	< 0.001*	3.61 (2.46, 4.76)	< 0.001*	0.62 (-0.42, 1.66)	0.423
Pain (VAS)	0.11 (-0.56, 0.79)	0.950	-0.26 (-1.00, 0.48)	0.477	-0.38 (-0.98, 0.23)	0.377	1.75 (0.94, 2.56)	< 0.001*	3.42 (2.57, 4.27)	< 0.001*	1.67 (0.75, 2.59)	< 0.001*
*HRQoL*												
Social function	0.91 (-2.13, 3.96)	0.950	0.77 (-3.87, 5.41)	0.950	-0.14 (-3.23, 2.95)	0.950	-0.32 (-4.01,3.36)	0.950	-1.41 (-6.77, 3.94)	0.950	-1.09 (-6.04, 3.86)	0.950
Mental health	-0.83 (-8.12, 6.46)	0.950	-1.51 (-6.09, 3.06)	0.950	-0.69 (-6.82, 5.44)	0.950	1.85 (-1.45, 5.15)	0.498	3.91 (-2.79, 10.61)	0.451	2.06 (-4.46, 8.58)	0.950
Social support	-0.57 (-3.87, 2.72)	0.950	-0.63 (-4.65, 3.39)	0.950	-0.06 (-2.94, 2.82)	0.950	-0.53 (-3.69, 2.63)	0.950	-1.03 (-5.74, 3.68)	0.950	-0.5 (-4.97, 3.97)	0.950
Pain	-0.09 (-2.41,2.24)	0.950	-1.06 (-5.55,3.44)	0.950	-0.97 (-5.18, 3.24)	0.950	2.74 (-1.84, 7.31)	0.424	4.41 (0.91, 7.92)	0.010*	1.68 (-1.75, 5.10)	0.676
PA function	-0.91 (-6.32,4.48)	1.000	-2.17 (-7.07,2.73)	1.000	-1.26 (-5.37,2.86)	1.000	3.85 (0.57,7.13)	0.385	5.79 (3.12,8.47)	< 0.010*	1.68 (-1.75, 5.10)	0.017*

MD; mean difference, CI; confidence intervals; 30CST, 30-s chair stand test; ALP, alkaline phosphatase; ALT, alanine transaminase; AST, aspartate transaminase; BMI, body mass index; FBG, fasting blood glucose; HDL-C, high-density lipoprotein cholesterol; HRQoL, health-related quality of life; LDL-C, low-density lipoprotein cholesterol; PA, physical activity; TC, total cholesterol; TG, triglycerides; TUG, timed up and go test. * denotes statistical significance (*p* < 0.05

Table 4 presents the group-by-time interaction effects. There were significant differences in TUG and the pain score between CON and HBCT at mid- (*p* = 0.003 − 0.002) and post-training (*p* < 0.001). At post-training, meaningful differences were detected in the pain score, FBG, TC, HDL-C, LDL-C, TG, total protein, AST and ALT between groups (*p* < 0.05), but not in ALP and all HRQoL’s dimensions, besides pain (*p* = 0.016).

**Table 4:** Group-by-time interaction effects

Variables	Time	MD (95% CI)	*p*	η^2^
BMI (kg/m^2^)	Pre	-0.40 (-2.79, 1.99)	0.739	0.001
	Mid	-1.14 (-3.73, 1.46)	0.385	0.027
	Post	-2.47 (-4.88, -0.06)	0.045*	0.055
FBG (mmol/L)	Pre	0.41 (-0.83, 1.64)	0.515	0.003
	Post	-1.42 (-2.70, -0.13)	0.031*	0.068
*Musculoskeletal Fitness*				
30CST (repetitions)	Pre	-1.84 (-3.07, -0.61)	0.054	0.117
	Mid	0.54 (-0.56, 1.64)	0.332	0.014
	Post	2.08 (0.98, 3.19)	< 0.001*	0.174
TUG (s)	Pre	1.23 (-0.12, 2.58)	0.072	0.047
	Mid	-1.99 (-3.29, -0.70)	0.003*	0.123
	Post	-3.08 (-4.49, -1.66)	< 0.001*	0.219
Pain (VAS)	Pre	-0.04 (-0.91, -0.83)	0.931	0.001
	Mid	-1.67 (-2.70, -0.65)	0.002*	0.169
	Post	-3.72 (-4.70, -2.74)	< 0.001*	0.459
*Blood Lipid Profile*				
TC (mmol/L)	Pre	0.34 (-0.21, 0.90)	0.218	0.022
	Post	-0.87 (-1.46, -0.28)	0.004*	0.115
HDL-C (mmol/L)	Pre	-0.09 (-0.21, 0.03)	0.170	0.027
	Post	0.25 (0.12, 0.37)	< 0.001*	0.187
LDL-C (mmol/L)	Pre	0.52 (-0.02, 1.05)	0.060	0.052
	Post	-0.56 (-1.03, -0.09)	0.020*	0.078
TG (mmol/L)	Pre	0.35 (-0.03, 0.03)	0.070	0.048
	Post	-0.48 (-0.78, -0.17)	0.003*	0.128
*Liver Function*				
Total protein (U/L)	Pre	-0.47 (-2.69, 1.76)	0.678	0.026
	Post	2.35 (0.67, 4.03)	0.007*	0.006
AST (U/L)	Pre	-2.86 (-8.22, 2.51)	0.292	0.016
	Post	-5.99 (-10.54, -1.45)	< 0.010*	0.093
ALT (U/L)	Pre	3.34 (-4.66, 11.35)	0.408	0.010
	Post	-8.89 (-17.54, -0.25)	0.044*	0.059
ALP (U.L)	Pre	-0.06 (-0.158, 0.04)	0.247	0.006
	Post	-0.08 (-0.17, 0.04)	0.143	0.009
*HRQoL*				
Social function	Pre	-3.86 (-7.84, 0.11)	0.057	0.053
	Mid	-2.63 (-6.12, 0.86)	0.138	0.032
	Post	-1.68 (-5.80, 2.44)	0.419	0.009
Mental health	Pre	2.22 (-7.29, 11.74)	0.642	0.003
	Mid	-0.46 (-9.85, 8.93)	0.923	0.000
	Post	-3.20 (-12.07, 5.67)	0.474	0.007
Social support	Pre	-1.01 (-4.93, 2.90)	0.607	0.003
	Mid	-1.05 (-4.78, 2.67)	0.574	0.004
	Post	-0.61 (-4.31,3.08)	0.742	0.001
Pain	Pre	0.69 (-3.55, 4.92)	0.746	0.011
	Mid	-2.13 (-6.51, 2.26)	0.336	0.013
	Post	-4.78 (-8.63, -0.93)	0.016*	0.083
Physical activity function	Pre	1.97 (-6.47, 10.40)	0.643	0.003
	Mid	-0.89 (-9.31, 7.53)	0.834	0.001
	Post	-6.00 (-13.02, 1.02)	0.093	0.041

MD; mean difference, CI; confidence intervals; 30CST, 30-s chair stand test; ALP, alkaline phosphatase; ALT, alanine transaminase; AST, aspartate transaminase; BMI, body mass index; FBG, fasting blood glucose; HDL-C, high-density lipoprotein cholesterol; LDL-C, low-density lipoprotein cholesterol; TC, total cholesterol; TG, triglycerides; TUG, timed up and go test. * denotes statistical significance (*p < 0.05)*

## Discussion

### Main findings

The present randomized controlled trial pointed to investigate the efficacy of a 3-month HBCT on numerous indicators related to KOA, cardiometabolic health, as well as mental health among individuals with overweight/obesity, T2DM and KOA. To our knowledge, this is the first pragmatic study evaluating the effects of such a home-based exercise protocol in this cohort. The current results indicated that an injury-free HBCT program meaningfully improved BMI, blood lipid profile, FBG, liver function, musculoskeletal fitness and HRQoL.

Despite the general recommendation of rehabilitation as a primary treatment for KOA, there is inconsistency in the standard approach to therapy programs. According to a study by Nguyen, Lefevre-Colau [51] focusing on the effectiveness and safety of strength training and exercise therapy, rehabilitation is widely advised and considered a crucial treatment for KOA. However, a gap needs to be addressed in improving the reasons for prescribing exercise. One promising approach to maximize the benefits of exercise is CT, which enhances cardiorespiratory and muscular fitness. CT can be advantageous for various chronic conditions, including KOA, as it involves repeatedly performing a series of exercises with minimal or no rest intervals [32]. It integrates muscle-strengthening exercises and bodyweight movements into a circuit fashion, aiming to maintain an elevated heart rate throughout the workout [33, 34]. CT may offer superior advantages compared to traditional aerobic exercise [35, 36]. Moreover, the relatively brief period allocated to each activity in cognitive therapy creates an environment that is favorable for group engagement, promoting enhanced commitment and cooperation among the individuals involved [32, 33, 36, 52]. Lowering body fat has been demonstrated as a positive outcome of CT, contributing to improved body composition [53]. The CT has the potential to more effectively stimulate the breakdown of fat tissue (lipolysis) compared to traditional aerobic training [54, 55].

### Blood lipids

In terms of the improvements in lipid homeostasis, the present outcomes are consistent with those reported in previous studies investigating CART in lipid homeostasis [56–61]. The beneficial changes in the lipid profile after exercise could be due to the involvement of larger muscle mass in CT. This could have caused a greater reduction in intramyocytic fat content and increased fatty acid oxidation capacity compared to CON. These positive effects may have caused an increase in the clearance of lipids from circulation [62, 63]. Physical exercise can increase the expression of PPAR and PGC-1 messenger RNAs, leading to improved metabolic flexibility and energy utilization in both muscle and adipose (fat) tissue as reported by a previous study [64]. In addition, the improvement in lipid metabolism following an exercise intervention may suggest enhanced cardiovascular health, as exercise is known to positively impact lipid levels by reducing LDL-C and increasing HDL-C. This particular improvement could potentially lower the risk of cardiovascular complications; however, the exact mechanism behind these changes in lipids is unknown, and thus further research is needed in this area [65]. Also, a recent meta-analysis examined the effect of aerobic and resistance training on lipid profiles in people with T2DM [66]. The findings of the included studies were varied, with two claiming that exercise had no impact on LDL-C, HDL-C, TC, or TG [67, 68], and one study reported exercise lower TC and altered HDL-C and LDL-C levels [69]. Other studies have shown a significant reduction in TG [70–72] and TC [73–77]; however no difference in LDL-c and HDL-c was found following combined aerobic and resistance training [74, 75]. The conflicts between these findings may be explained by the difference in the exercise intensity, volume and the baseline participants’ values, which were not significantly changed after the exercise intervention [58].

### Liver function

In the present study, liver function parameters showed exceptional reductions in HBCT compared to CON, indicating that exercise improved blood flow to the liver, decreased liver inflammation, reduced fat in the liver, and reduced whole-body fat [78, 79]. Exercise increases blood flow throughout the body, including the liver [80], and therefore this enhanced blood flow delivers oxygen and nutrients to the liver, facilitating its metabolic processes and supporting overall liver health [81]. Improved oxygenation and nutrient supply can reduce liver injury and normalize ALT and AST levels [82]. In line with our study, Słomko, Zalewska [83] conducted a systematic review and meta-analysis of 15 RCTs involving 740 patients with metabolic-associated fatty liver disease; most of the included studies involved overweight and obese patients with underlying T2DM. It was found that all types of aerobic exercise protocols significantly decreased ALT levels and improved the metabolically associated fatty liver disease compared with the control group [84]. On the other hand, other study found no significant difference in liver function after the intervention [85]. However, it is worth noting that the extent of improvement may vary depending on factors, such as the baseline liver function, exercise intensity, and duration as well as the overall lifestyle habits of the patients.

### Glucose control

In the current study, we found a significant decrease in FBG in the intervention group, which is in accordance with previous studies that conducted a combination of aerobic and resistance training [61, 71, 73, 86–92]. This finding could be attributed to the improvement of insulin-stimulated glucose transport in skeletal muscle and increased tissue exposure to insulin and glucose [93–96]. In other studies, overweight and sedentary patients with underlying T2DM have shown an improvement in insulin action after 4 to 8 months of physical training [97, 98]. The observed enhancement in the glucose tolerance in the T2DM patients in the intervention group could be due to an increase in the glucose clearance rate associated with an increase in muscle blood flow as well as an improvement in the body’s capacity to absorb glucose [99]. In contrast, a previous study of elderly patients with T2DM did not find any change in FBG after 16 weeks of combined aerobic and resistance training [99]. Other studies reported no difference in FBG following the exercise [74, 75]. The inconsistencies in these studies compared to the present study could be due to the physiological complexity involved, the small sample size, and the variations in exercise training procedures.

### Anthropometry

As for BMI, significant reductions were detected in HBCT which is in line with previous studies [71, 73], suggesting that exercise increases energy expenditure resulting in a calorie deficit and contributing to weight loss. A similar finding was also found in a study that demonstrated a substantial decrease in BMI after six months of home-based combined aerobic and resistance training among T2DM patients [100]. This finding may be attributed to the HBCT as well as methodological considerations, high levels of compliance and retention among the participants [58, 74, 87, 100–103]. Furthermore, it is unlikely that any changes in diet or exercise routines that existed independent of the intervention had an influence on our results. In the present study, no changes in dietary patterns or outside-of-the-study physical activity were found in the participants’ logbook, which was evaluated at each visit.

### Musculoskeletal fitness

Concerning musculoskeletal fitness indices, the scores for 30CST and TUG demonstrated significant elevations following HBCT that combined aerobic and resistance training consistent with previous studies in patients with T2DM [14, 73, 88, 91, 104, 105]. This finding implies that exercise may improve muscular fitness and functional capacity in KOA patients. Contrastingly, another study failed to identify a notable difference in TUG performance [106]. Similarly, a recent systematic review and meta-analysis revealed the absence of significant improvements in functional performance based on 30CST and TUG scores [107]. This discrepancy may be ascribed to variations in participants’ baseline fitness levels, inadequate intervention duration to provoke discernible changes, potential methodological constraints, or the necessity for more specific and targeted exercise interventions. In general, positive musculoskeletal adaptations to CT-like programs are critical for this cohort, since people with excessive weight, impaired glucose metabolism and KOA are very likely to experience poor functionality, numerous physical limitations and limited independence when performing activities of daily living [108, 109]. Hence, time-efficient, integrated exercise training solutions may be important tools for inducing noticeable improvements in muscular fitness, flexibility, mobility, agility, dynamic balance and functionality as previously reported [23, 26].

### Health-related quality of life

Regarding alterations in Health-Related Quality of Life HRQoL, meaningful enhancements were observed in specific dimensions related to KOA. More specifically, the physical activity function and pain scores for HBCT were similar to previous studies [14, 91]. These findings could be possibly due to the improvement in muscular strength which might likely decrease knee pain and improve walking ability, which subsequently, improves the HRQoL of the patients in the current study. Another research recruiting elderly T2DM patients reported that 16 weeks of combined aerobic and resistance training improved both walking capacity and HRQoL [99]. The present improvements in HRQoL, musculoskeletal fitness, and pain among patients with overweight/obesity, T2DM and KOA following HBCT may support the prescribed training structure and configuration that combine aerobic- and resistance-based activities for enhancing both cardiorespiratory and neuromuscular conditioning in a time-efficient manner. This is certainly relevant for people with T2DM who have comorbidities that might restrict traditional gym-based exercise circuits or who do not feel comfortable in a gym and/or hospital environment.

### Pain

Regarding the pain score, a significant improvement was noted in HBCT compared to CON. This finding could be possibly due to the improvement observed in lower body muscular strength and endurance, functionality, and mobility following the exercise intervention. Such an observation underlines the key role of exercise in lowering pain, increasing range of motion and flexibility as well as strengthening the knee joint muscles. In agreement with our study, a recent systematic review and meta-analysis incorporated seven trials with 346 patients diagnosed with KOA, revealing a significant reduction in the pain score following a CT program [107].

### Comorbidity

However, comorbidity can pose additional difficulties in implementing exercise therapy for KOA patients [110]. According to the previous study, it was found that the presence of comorbidities has major effects on the prognosis [111] and may affect treatments; thus, it should be taken into concertation [112]. Unfortunately, no evidence-supported method is available for treating individuals with both KOA and additional health conditions [113]. The current KOA guidelines do not provide specific recommendations for exercise adaptations related to comorbidities [114–116]. In certain cases, merging multiple treatment plans for different diseases may be infeasible because a particular therapy could interfere with the normal progression of a coexisting condition or have adverse interactions with another medication [117]. Moreover, exercise is rarely recommended for elderly individuals with KOA and serious underlying comorbidities. They frequently discontinue the treatment or receive inadequate care as therapists may reduce the intensity of the exercise to an insufficient level. Consequently, no evidence-based exercise regimen is ideal for managing overweight individuals with KOA and T2DM. Therefore, the present study provides important insights into the positive role of a real-world exercise solution, integrating cardiorespiratory and neuromuscular stimulus into a single, time-efficient session for a cohort representing the vast majority of adults in the Western world [118].

### Strengths and limitations

The current findings cannot be generalized to people of other ethnic groups, ages, and BMI ranges. Nevertheless, the recruitment of older adults with impaired cardiometabolic and musculoskeletal health aims to represent an important cohort. In addition, this randomized controlled trial did not examine various cardiovascular risk factors associated with metabolic dysregulation, such as resting blood pressure, glucose homeostasis, redox status, and cardiorespiratory fitness. However, the conduction of a pragmatic study in an unsupervised, home-based setting during the COVID-19 pandemic may provide insights into time-efficient and feasible exercise approaches under real conditions for people demonstrating insufficient physical activity levels while being impacted by the most common lifestyle-related chronic diseases. Furthermore, the lack of additional psychometrics, such as exercise enjoyment and affective valence did not provide data related to the potential association between pleasure and high adherence (98.6%) and attendance (91.7%) rates, which were relatively high in comparison to the earlier studies [70, 71, 88, 91, 92, 119], especially since 80% of patient retention at the end of the intervention was considered to be very high [120, 121]. Also, the dose-response effects investigation between HBCT and various physiological, psychological, biochemical and hormonal indicators among people with overweight/obesity, KOA and T2DM may be an important direction in a future research attempt in this area as previously articulated [38]. Finally, the lack of a reliable and valid questionnaire assessing physical activity and caloric intake is an additional limitation, since only self-report information was collected through an interview in order to ensure that participants did not change their habits throughout the intervention.

## Conclusion

The findings from our study demonstrate compelling improvements in lipid profiles, musculoskeletal fitness, and OA-related QoL among overweight or obese older adults with T2DM and KOA following a 3-month HBCT. Our pragmatic approach highlights significant reductions in TC, TG, LDL-C, aminotransferase levels, alanine aminotransferase, FBG, and knee pain. Notably, HBCT also led to meaningful increases in HDL-C, lower body muscular strength, endurance, functional capacity, mobility, and HRQoL. These outcomes underscore the potential public health impact of a practical, injury-free exercise solution integrating bodyweight and resistance-based activities in a home-based setting. Our results suggest that HBCT may serve as a supplementary real-world exercise strategy for individuals characterised by sedentary lifestyles, excess weight, and metabolic dysregulation, such as those with T2DM and KOA. In summary, the targeted improvements observed in lipid metabolism, musculoskeletal health, and overall well-being among this at-risk population demonstrate the efficacy and feasibility of HBCT as an accessible and impactful exercise approach. These findings provide valuable insights for medical and exercise professionals seeking evidence-based interventions to address the complex health challenges faced by older adults with comorbidities such as overweight/obesity, T2DM, and KOA [122].

## Data Availability

All data generated or analysed during this study are included in this published article [and its supplementary information files].

## References

[1] Abarca-Gómez L (2017). Worldwide trends in body-mass index, underweight, overweight, and obesity from 1975 to 2016: a pooled analysis of 2416 population-based measurement studies in 128· 9 million children, adolescents, and adults. Lancet.

[2] Afshin A, Reitsma MB, Murray CJ (2017). Health effects of overweight and obesity in 195 countries. N Engl J Med.

[3] Gregg EW, Shaw JE. Global health effects of overweight and obesity. Mass Medical Soc; 2017. pp. 80–1. 10.1056/NEJMe1706095 28604226

[4] Zullig LL et al. The association of comorbid conditions with patient-reported outcomes in veterans with hip and knee osteoarthritis. 2015. 34(8): p. 1435–41. 10.1007/s10067-014-2707-y PMC4782794 24916605

[5] Shin D, Metabolism. Association between metabolic syndrome, radiographic knee osteoarthritis, and intensity of knee pain: results of a national survey. 2014. 99(9): p. 3177–83. 10.1210/jc.2014-1043 24780047

[6] Li H et al. *Metabolic syndrome and components exacerbate osteoarthritis symptoms of pain, depression and reduced knee function* 2016. 4(7). 10.21037/atm.2016.03.48 PMC4842398 27162783

[7] Anderson JJ. And D.T.J.A.j.o.e. Felson, factors associated with osteoarthritis of the knee in the first national Health and Nutrition Examination Survey (HANES I) evidence for an association with overweight, race, and physical demands of work. 1988. 128(1): p. 179–89. 10.1093/oxfordjournals.aje.a114939 3381825

[8] Nieves-Plaza M (2013). Association of hand or knee osteoarthritis with diabetes mellitus in a population of hispanics from Puerto Rico. JCR: J Clin Rheumatol.

[9] Schett G (2013). Diabetes is an independent predictor for severe osteoarthritis: results from a longitudinal cohort study. Diabetes Care.

[10] Control CfD, Prevention (2008). Arthritis as a potential barrier to physical activity among adults with diabetes–United States, 2005 and 2007. MMWR Morb Mortal Wkly Rep.

[11] Andersen H (2004). Muscle strength in type 2 diabetes. Diabetes.

[12] Park SW (2006). Decreased muscle strength and quality in older adults with type 2 diabetes: the health, aging, and body composition study. Diabetes.

[13] Tajiri Y (2010). Reduction of skeletal muscle, especially in lower limbs, in Japanese type 2 diabetic patients with insulin resistance and cardiovascular risk factors. Metab Syndr Relat Disord.

[14] Maiorana A (2002). Combined aerobic and resistance exercise improves glycemic control and fitness in type 2 diabetes. Diabetes Res Clin Pract.

[15] Lord SR (2002). Sit-to-stand performance depends on sensation, speed, balance, and psychological status in addition to strength in older people. Journals Gerontol Ser A: Biol Sci Med Sci.

[16] Neogi T (2013). The epidemiology and impact of pain in osteoarthritis. Osteoarthr Cartil.

[17] Goodman SM (2020). Patients’ perspectives of outcomes after total knee and total hip arthroplasty: a nominal group study. BMC Rheumatol.

[18] Enright PL (2003). The six-minute walk test. Respir Care.

[19] Frontera WR et al. Muscle fiber size and function in elderly humans: a longitudinal study. 2008. 105(2): p. 637–42. 10.1152/japplphysiol.90332.2008 PMC2519941 18556434

[20] Beavers KM et al. Effects of total and regional fat loss on plasma CRP and IL-6 in overweight and obese, older adults with knee osteoarthritis. 2015. 23(2): p. 249–56. 10.1016/j.joca.2014.11.005 PMC4304884 25450847

[21] Bennell KL, Hinman RSJJoS, Sport Mi (2011). Rev Clin Evid Exerc Osteoarthr hip knee.

[22] Kercher VM (2023). 2023 Fitness Trends from around the Globe. ACSMs Health Fit J.

[23] Batrakoulis A et al. Hybrid-type, multicomponent interval training upregulates musculoskeletal fitness of adults with overweight and obesity in a volume-dependent manner: a 1-year dose-response randomised controlled trial. Eur J Sport Sci, 2022: p. 1–12. 10.1080/17461391.2021.2025434 34974824

[24] Batrakoulis A (2022). Comparative efficacy of 5 Exercise types on Cardiometabolic Health in overweight and obese adults: a systematic review and network Meta-analysis of 81 randomized controlled trials. Circ Cardiovasc Qual Outcomes.

[25] Batrakoulis A, Fatouros IG. Psychological adaptations to high-intensity interval training in overweight and obese adults: a topical review. Sports (Basel), 2022. 10(5). 10.3390/sports10050064 PMC9148041 35622474

[26] Batrakoulis A (2021). Hybrid neuromuscular training promotes musculoskeletal adaptations in inactive overweight and obese women: a training-detraining randomized controlled trial. J Sports Sci.

[27] Batrakoulis A, Jamurtas AZ, Fatouros IG (2021). High-intensity interval training in metabolic diseases: physiological adaptations. ACSM’s Health Fit J.

[28] Batrakoulis A et al. Hybrid Neuromuscular Training Improves Cardiometabolic Health and alters Redox Status in inactive overweight and obese women: a Randomized Controlled Trial. Antioxid (Basel), 2021. 10(10). 10.3390/antiox10101601 PMC8533161 34679738

[29] Batrakoulis A (2018). High intensity, circuit-type integrated neuromuscular training alters energy balance and reduces body mass and fat in obese women: a 10-month training-detraining randomized controlled trial. PLoS ONE.

[30] Gilyana M, Batrakoulis A, Zisi V. Physical activity, body image, and Emotional Intelligence Differences in adults with overweight and obesity. Diseases, 2023. 11(2). 10.3390/diseases11020071 PMC10204461 37218884

[31] Batrakoulis A (2020). High-intensity interval neuromuscular training promotes exercise behavioral regulation, adherence and weight loss in inactive obese women. Eur J Sport Sci.

[32] Bocalini DS (2012). Effects of circuit-based exercise programs on the body composition of elderly obese women. Clin Interv Aging.

[33] Miller MB et al. The effect of a short-term high-intensity circuit training program on work capacity, body composition, and blood profiles in sedentary obese men: a pilot study BioMed research international, 2014. 2014. 10.1155/2014/191797 PMC3953517 24707476

[34] Romero-Arenas S (2013). Effects of high-resistance circuit training in an elderly population. Exp Gerontol.

[35] Balachandran A (2014). High-speed circuit training vs hypertrophy training to improve physical function in sarcopenic obese adults: a randomized controlled trial. Exp Gerontol.

[36] Kim H-J (2014). Effects of vitamin D supplementation and circuit training on indices of obesity and insulin resistance in T2D and vitamin D deficient elderly women. J Exerc Nutr Biochem.

[37] Newsome AM (2024). 2024 ACSM Worldwide Fitness trends: future directions of the Health and Fitness Industry. ACSM’s Health Fit J.

[38] Batrakoulis A (2019). Dose-response effects of high-intensity interval neuromuscular exercise training on weight loss, performance, health and quality of life in inactive obese adults: study rationale, design and methods of the DoIT trial. Contemp Clin Trials Commun.

[39] De Groot V (2003). How to measure comorbidity: a critical review of available methods. J Clin Epidemiol.

[40] Huang Y-q (2014). Charlson comorbidity index helps predict the risk of mortality for patients with type 2 diabetic nephropathy. J Zhejiang Univ Sci B.

[41] Charlson ME (1987). A new method of classifying prognostic comorbidity in longitudinal studies: development and validation. J Chronic Dis.

[42] Chen CK et al. Combined effects of Lignosus rhinocerotis supplementation and resistance training on isokinetic muscular strength and power, anaerobic and aerobic fitness level, and immune parameters in young males. Int J Prev Med, 2016. 7. 10.4103/2008-7802.190604 PMC5036278 27833721

[43] Borg GA (1982). Psychophysical bases of perceived exertion. Med Sci Sports Exerc.

[44] Friedewald WT, Levy RI, Fredrickson DS (1972). Estimation of the concentration of low-density lipoprotein cholesterol in plasma, without use of the preparative ultracentrifuge. Clin Chem.

[45] Jones CJ, Rikli RE, Beam WC (1999). A 30-s chair-stand test as a measure of lower body strength in community-residing older adults. Res Q Exerc Sport.

[46] Podsiadlo D, Richardson S (1991). The timed up & go: a test of basic functional mobility for frail elderly persons. J Am Geriatr Soc.

[47] Abdul Kadir A et al. *Adaptation and validation of the malay version of the osteoarthritis knee and hip quality of life questionnaire among knee osteoarthritis patients* BioMed research international, 2018. 2018. 10.1155/2018/4329751 PMC6000845 29955601

[48] Rat AC (2005). OAKHQOL: a new instrument to measure quality of life in knee and hip osteoarthritis. J Clin Epidemiol.

[49] Delgado DA (2018). Validation of Digital Visual Analog Scale Pain Scoring with a traditional paper-based Visual Analog Scale in adults. J Am Acad Orthop Surg Glob Res Rev.

[50] Lakens D (2013). Calculating and reporting effect sizes to facilitate cumulative science: a practical primer for t-tests and ANOVAs. Front Psychol.

[51] Nguyen C et al. Rehabilitation (exercise and strength training) and osteoarthritis: a critical narrative review. 2016. 59(3): p. 190–5. 10.1016/j.rehab.2016.02.010 27155923

[52] Nordgren B (2015). An outsourced health-enhancing physical activity programme for people with rheumatoid arthritis: exploration of adherence and response. Rheumatology.

[53] Bocalini DS et al. Effects of circuit-based exercise programs on the body composition of elderly obese women. 2012. 7: p. 551. 10.2147/CIA.S33893 PMC3526879 23271901

[54] Romero-Arenas S et al. Effects of high-resistance circuit training in an elderly population. 2013. 48(3): p. 334–40. 10.1016/j.exger.2013.01.007 23352954

[55] Balachandran A et al. High-speed circuit training vs hypertrophy training to improve physical function in sarcopenic obese adults: a randomized controlled trial. 2014. 60: p. 64–71. 10.1016/j.exger.2014.09.016 25281504

[56] Sabouri M (2021). Inflammatory, antioxidant and glycemic status to different mode of high-intensity training in type 2 diabetes mellitus. Mol Biol Rep.

[57] Zarei M et al. The effect of combined resistance aerobic exercise training on concentrations of asprosin and complement C1q tumor necrosis factor-related protein-1 in men with type 2 diabetes. Sport Sci Health, 2021: p. 1–9.

[58] Magalhães JP (2020). Impact of combined training with different exercise intensities on inflammatory and lipid markers in type 2 diabetes: a secondary analysis from a 1-year randomized controlled trial. Cardiovasc Diabetol.

[59] Al-Mhanna SB et al. Impact of various types of Exercise on lipid metabolism in patients with type 2 diabetes and concurrent Overweight/Obesity: a narrative review. Ann Appl Sport Sci, 2024. 12(1).

[60] Al-Mhanna SB et al. Impact of Exercise on High-Density Lipoprotein Cholesterol in adults with overweight and obesity: a narrative review. Ann Appl Sport Sci, 2024. 12(1).

[61] Al-Mhanna SB (2023). Effects of combined aerobic exercise and diet on cardiometabolic health in patients with obesity and type 2 diabetes: a systematic review and meta-analysis. BMC Sports Sci Med Rehabil.

[62] Wang Y, Xu D (2017). Effects of aerobic exercise on lipids and lipoproteins. Lipids Health Dis.

[63] Kim HJ, Lee JS, Kim CK (2004). Effect of exercise training on muscle glucose transporter 4 protein and intramuscular lipid content in elderly men with impaired glucose tolerance. Eur J Appl Physiol.

[64] Ruschke K (2010). Gene expression of PPARγ and PGC-1α in human omental and subcutaneous adipose tissue is related to insulin resistance markers and mediates beneficial effects of physical training. Eur J endocrinology/European Federation Endocr Soc.

[65] Earnest CP et al. *Maximal estimated cardiorespiratory fitness, cardiometabolic risk factors, and metabolic syndrome in the aerobics center longitudinal study*. in *Mayo Clinic Proceedings*. 2013. Elsevier. 10.1016/j.mayocp.2012.11.006 PMC3622904 23391253

[66] De Nardi AT (2018). High-intensity interval training versus continuous training on physiological and metabolic variables in prediabetes and type 2 diabetes: a meta-analysis. Diabetes Res Clin Pract.

[67] Terada T (2013). Feasibility and preliminary efficacy of high intensity interval training in type 2 diabetes. Diabetes Res Clin Pract.

[68] Maillard F (2016). High-intensity interval training reduces abdominal fat mass in postmenopausal women with type 2 diabetes. Diabetes Metab.

[69] Mitranun W (2014). Continuous vs interval training on glycemic control and macro-and microvascular reactivity in type 2 diabetic patients. Scand J Med Sci Sports.

[70] Yavari A (2012). Effect of aerobic exercise, resistance training or combined training on glycaemic control and cardio-vascular risk factors in patients with type 2 diabetes. Biology Sport.

[71] Gibbs BB (2012). A randomized trial of exercise for blood pressure reduction in type 2 diabetes: effect on flow-mediated dilation and circulating biomarkers of endothelial function. Atherosclerosis.

[72] Duncan GE (2003). Exercise training, without weight loss, increases insulin sensitivity and postheparin plasma lipase activity in previously sedentary adults. Diabetes Care.

[73] Lambers S (2008). Influence of combined exercise training on indices of obesity, diabetes and cardiovascular risk in type 2 diabetes patients. Clin Rehabil.

[74] Annibalini G et al. Concurrent aerobic and resistance training has anti-inflammatory effects and increases both plasma and leukocyte levels of IGF-1 in late middle-aged type 2 diabetic patients Oxidative medicine and cellular longevity, 2017. 2017. 10.1155/2017/3937842 PMC5497609 28713486

[75] Mann S, Beedie C, Jimenez A (2014). Differential effects of aerobic exercise, resistance training and combined exercise modalities on cholesterol and the lipid profile: review, synthesis and recommendations. Sports Med.

[76] Hansen D (2009). Continuous low-to moderate-intensity exercise training is as effective as moderate-to high-intensity exercise training at lowering blood HbA1c in obese type 2 diabetes patients. Diabetologia.

[77] Gordon LA (2008). Effect of exercise therapy on lipid profile and oxidative stress indicators in patients with type 2 diabetes. BMC Complement Altern Med.

[78] Thorp A, Stine JG (2020). Exercise as medicine: the impact of exercise training on nonalcoholic fatty liver disease. Curr Hepatol Rep.

[79] Hughes A (2021). Exercise training reverses gut dysbiosis in patients with biopsy-proven nonalcoholic steatohepatitis: a proof of concept study. Clin Gastroenterol Hepatol.

[80] Delp MD (2001). Exercise increases blood flow to locomotor, vestibular, cardiorespiratory and visual regions of the brain in miniature swine. J Physiol.

[81] Shephard RJ, Johnson N (2015). Effects of physical activity upon the liver. Eur J Appl Physiol.

[82] Tyerman J, et al. Lewis’s Medical-Surgical Nursing in Canada-E-Book: Assessment and Management of Clinical Problems. Elsevier Health Sciences; 2022.

[83] Słomko J (2021). Evidence-based aerobic exercise training in metabolic-associated fatty liver disease: systematic review with meta-analysis. J Clin Med.

[84] Chen Z (2020). Role of oxidative stress in the pathogenesis of nonalcoholic fatty liver disease. Free Radic Biol Med.

[85] Oliveira VNd et al. *The effect of different training programs on antioxidant status, oxidative stress, and metabolic control in type 2 diabetes* Applied Physiology, Nutrition, and Metabolism, 2012. 37(2): pp. 334–344. 10.1139/h2012-004 22458821

[86] Bellini A (2021). The effect of different postprandial exercise types on glucose response to breakfast in individuals with type 2 diabetes. Nutrients.

[87] Church TS (2010). Effects of aerobic and resistance training on hemoglobin A1c levels in patients with type 2 diabetes: a randomized controlled trial. JAMA.

[88] Dunstan DW (1998). Effects of a short-term circuit weight training program on glycaemic control in NIDDM. Diabetes Res Clin Pract.

[89] Earnest CP (2014). Aerobic and strength training in concomitant metabolic syndrome and type 2 diabetes. Med Sci Sports Exerc.

[90] Kadoglou N (2013). The differential anti-inflammatory effects of exercise modalities and their association with early carotid atherosclerosis progression in patients with type 2 diabetes. Diabet Med.

[91] Scheer AS (2020). The effects of water-based exercise training in people with type 2 diabetes. Med Sci Sports Exerc.

[92] Tan S, Li W, Wang J (2012). Effects of six months of combined aerobic and resistance training for elderly patients with a long history of type 2 diabetes. J Sports Sci Med.

[93] Henriksen EJ (2002). Invited review: effects of acute exercise and exercise training on insulin resistance. J Appl Physiol.

[94] Maiorana A (2001). The effect of combined aerobic and resistance exercise training on vascular function in type 2 diabetes. J Am Coll Cardiol.

[95] Maiorana A et al. Effect of aerobic and resistance exercise training on vascular function in heart failure. Am J Physiol Heart Circ Physiol, 2000. 279(4): p. H1999-H2005. 10.1152/ajpheart.2000.279.4.H1999 11009490

[96] Saltin B. Skeletal muscle adaptability: significance for metabolism and performance. Skeletal muscle; 1983.

[97] Slentz CA (2009). Effects of exercise training intensity on pancreatic β-cell function. Diabetes Care.

[98] Tokmakidis SP (2004). The effects of a combined strength and aerobic exercise program on glucose control and insulin action in women with type 2 diabetes. Eur J Appl Physiol.

[99] Tessier D (2000). Effects of aerobic physical exercise in the elderly with type 2 diabetes mellitus. Arch Gerontol Geriatr.

[100] Ferrer-García JC et al. *Benefits of a home-based physical exercise program in elderly subjects with type 2 diabetes mellitus* Endocrinología y Nutrición (English Edition), 2011. 58(8): pp. 387–394. 10.1016/j.endonu.2011.05.010 21816692

[101] Zarei M (2021). The effect of combined resistance aerobic exercise training on concentrations of asprosin and complement C1q tumor necrosis factor-related protein-1 in men with type 2 diabetes. Sport Sci Health.

[102] Sabouri M (2021). Inflammatory, antioxidant and glycemic status to different mode of high-intensity training in type 2 diabetes mellitus. Mol Biol Rep.

[103] Cuff DJ (2003). Effective exercise modality to reduce insulin resistance in women with type 2 diabetes. Diabetes Care.

[104] Christos ZE (2009). Lipoprotein profile, glycemic control and physical fitness after strength and aerobic training in post-menopausal women with type 2 diabetes. Eur J Appl Physiol.

[105] Larose J. *The effect of exercise training on physical fitness in type 2 diabetes mellitus*. 2009, University of Ottawa (Canada).

[106] Skou ST et al. The efficacy of 12 weeks non-surgical treatment for patients not eligible for total knee replacement: a randomized controlled trial with 1-year follow-up Osteoarthritis and cartilage, 2015. 23(9): pp. 1465–1475. 10.1016/j.joca.2015.04.021 25937024

[107] Al-Mhanna SB, et al. Effects of Circuit Training on patients with knee osteoarthritis: a systematic review and Meta-analysis. Healthcare. MDPI; 2022. 10.3390/healthcare10102041 PMC9601599 36292488

[108] Pataky Z (2014). Effects of obesity on functional capacity. Obes (Silver Spring).

[109] Warburton DE, Gledhill N, Quinney A (2001). Musculoskeletal fitness and health. Can J Appl Physiol.

[110] de Rooij M et al. Restrictions and contraindications for exercise therapy in patients with hip and knee osteoarthritis and comorbidity. 2013. 18(2): p. 101–11.

[111] van Dijk GM et al. Prognosis of limitations in activities in osteoarthritis of the hip or knee: a 3-year cohort study. 2010. 91(1): p. 58–66. 10.1016/j.apmr.2009.08.147 20103397

[112] Boyd CM et al. Clinical practice guidelines and quality of care for older patients with multiple comorbid diseases: implications for pay for performance. 2005. 294(6): p. 716–24. 10.1001/jama.294.6.716 16091574

[113] Lugtenberg M et al. Current guidelines have limited applicability to patients with comorbid conditions: a systematic analysis of evidence-based guidelines. 2011. 6(10): p. e25987. 10.1371/journal.pone.0025987 PMC3197602 22028802

[114] Fernandes L et al. EULAR recommendations for the non-pharmacological core management of hip and knee osteoarthritis 2013. 72(7): pp. 1125–1135. 10.1136/annrheumdis-2012-202745 23595142

[115] Peter W et al. Physiotherapy in hip and knee osteoarthritis: development of a practice guideline concerning initial assessment. Treatment and evaluation 2011. 36(3). 22113602

[116] Zhang W et al. OARSI recommendations for the management of hip and knee osteoarthritis: part III: changes in evidence following systematic cumulative update of research published through January 2009. 2010. 18(4): p. 476–99. 10.1016/j.joca.2010.01.013 20170770

[117] Van Weel C, Schellevis FGJL. *Comorbidity and guidelines: conflicting interests* 2006. 367(9510): pp. 550–550. 10.1016/S0140-6736(06)68198-1 16488782

[118] Colberg SR (2010). Exercise and type 2 diabetes: the American College of Sports Medicine and the American Diabetes Association: joint position statement. Diabetes Care.

[119] Sigal RJ (2007). Effects of aerobic training, resistance training, or both on glycemic control in type 2 diabetes: a randomized trial. Ann Intern Med.

[120] Otterman NM (2011). An exercise programme for patients with diabetic complications: a study on feasibility and preliminary effectiveness. Diabet Med.

[121] Lohmann H, Siersma V, Olivarius NF (2010). Fitness consultations in routine care of patients with type 2 diabetes in general practice: an 18-month non-randomised intervention study. BMC Fam Pract.

[122] Batrakoulis A, Jamurtas AZ, Fatouros IG (2022). Exercise and type II diabetes mellitus: a brief guide for exercise professionals. Strength Cond J.

